# The Effect of DHA Supplementation on Cognition in Patients with Bipolar Disorder: An Exploratory Randomized Control Trial

**DOI:** 10.3390/nu12030708

**Published:** 2020-03-06

**Authors:** Valentina Ciappolino, Giuseppe DelVecchio, Cecilia Prunas, Angela Andreella, Livio Finos, Elisabetta Caletti, Francesca Siri, Alessandra Mazzocchi, Andrea Botturi, Stefano Turolo, Carlo Agostoni, Paolo Brambilla

**Affiliations:** 1 Fondazione IRCCS Ca’ Granda-Ospedale Maggiore Policlinico, Department of Neurosciences and Mental Health, 20122 Milan, Italy; valentina.ciappolino@policlinico.mi.it (V.C.); Cecilia.prunas@policlinico.mi.it (C.P.); francescamarzia.siri@policlinico.mi.it (F.S.); 2 Department of Pathophysiology and Transplantation, University of Milan, 20122 Milan, Italy; 3 Department of Statistical Sciences, University of Padua, 35100 Padua, Italy; angela.andreella@phd.unipd.it; 4 Department of Developmental Psychology and Socialization, University of Padua, 35100 Padua, Italy; livio.finos@unipd.it; 5 Department of Clinical Sciences and Community Health, University of Milan, 20122 Milan, Italy; alessandra.mazzocchi1@gmail.com (A.M.); carlo.agostoni@unimi.it (C.A.); 6 SINGEP (Italian Society of pediatric Gastroenterology, Hepatology and Nutrition), Via Libero Temolo 4 (Torre U8), 20126 Milan, Italy; 7 Neurologic Clinic, Fondazione IRCCS Istituto neurologico Carlo Besta, 20133 Milan, Italy; andrea.botturi@istituto-besta.it; 8 Fondazione IRCCS Ca’ Granda-Ospedale Maggiore Policlinico, Pediatric Nephrology, Dialysis and Transplant Unit, 20122 Milan, Italy; stefano.turolo@policlinico.mi.it; 9 Pediatric Intermediate Care Unit, Fondazione IRCCS Ca’ Granda Ospedale Maggiore Policlinico, 20122 Milan, Italy

**Keywords:** bipolar disorder, docosahexaenoic acid, ω-3 long chain polyunsaturated fatty acids, cognitive functions

## Abstract

Bipolar disorder (BD) is a severe mental disorder with a wide range of cognitive deficits, both in the euthymic and acute phase of the disease. Interestingly, in recent years, there has been a growing interest in investigating the impact of ω-3 polyunsaturated fatty acids on cognition in BD. In this context, the aim of this study is to evaluate the effect of docosahexaenoic acid (C22:6 ω-3, DHA) supplementation on cognitive performances in euthymic BD patients. This is an exploratory, single-centre, double-blind randomized controlled trial evaluating 12 weeks DHA supplementation (1250 mg daily) vs. a placebo (corn oil) in 31 euthymic BD patients compared to 15 healthy controls (HCs) on cognitive functions, assessed by the Brief Assessment of Cognition in Affective Disorder (BAC-A). Plasma levels of DHA were measured. After 12 weeks of treatment, no significant group differences were observed in all neuropsychological tests between the four groups, except for the emotion inhibition test, where HCs with DHA had higher scores compared to either BD with DHA (*z* = 3.9, *p* = 0.003) or BD with placebo (*t* = 3.7, *p* = 0.005). Although our results showed that DHA could be effective for ameliorating cognition in healthy subjects, future studies are still needed to clarify the impact of DHA on cognition in BD.

## 1. Introduction

Bipolar disorder (BD) is a severe mental disorder characterized by extreme mood swings, including episodes of mania (or hypomania) and major depression, interspersed with periods of euthymia [1], which is often associated with reduced life expectancy and with the development of work and psychosocial disabilities [2]. Moreover, it has been reported that BD patients (from around 40% to 60%), showed a wide range of cognitive deficits, especially in verbal and visual memory as well as executive functions [3]. Notably, since BD is a mental disorder with a high heritability, it has been found that also unaffected first-degree relatives and the offspring of patients with BD showed a mild cognitive dysfunction [4], ultimately pointing towards the hypothesis that cognitive dysfunctions could be considered as putative endophenotypes for BD [5,6]. Indeed, many studies have consistently reported the presence of cognitive deficits both during acute and euthymic phases [4,5] as well as in premorbid stages, before illness onset [7], ultimately suggesting that they can be considered as trait-related markers of the disorder. Furthermore, after the end of an acute episode, either manic or depressive, a significant number of patients do not recover their prior level of cognitive performance, therefore resulting in reduced psychosocial functioning [5], which is not surprising since neurocognitive abilities have been found to be consistently associated with social, work, and global functioning deficits [5]. Therefore, based on this evidence, in the last decade, there has been growing interest in the investigation of the impact of both pharmacological and non-pharmacological treatments on cognition in BD, with, however, inconclusive results [8]. Specifically, for pharmacological treatments, available evidence showed that many different drugs, including lurasidone, vortioxetine and modafinil, seem to be efficacious in ameliorating cognitive deficits in BD patients [9,10,11].

With regards to non-pharmacological strategies, recent studies focused their attention on the study of the so-called “functional nutrients”, which are a group of nutrients and dietary supplementations that could exert an ameliorating effect on cognition [12]. Among these nutrients, ω-3 polyunsaturated fatty acids (ω-3 PUFAs), due to their pleiotropic properties, have been proposed as therapeutic supplements for a wide range of diseases, including arteriosclerosis, cancer, diabetes, hypertension, arthritis, dementia, psychiatric disorders and some autoimmune diseases [13]. Focusing on brain health, there is accumulating evidence of the neuroprotective functions of omega-3 long-chain polyunsaturated fatty acids (ω3 LC-PUFAs), which represent 20% of brain weight, docosahexaenoic acid (DHA, C22:6 ω-3) and arachidonic acid (AA, C20:4 ω-6) are the most abundant [14]. DHA, which is the most important ω-3 LC-PUFAs together with eicosapentaenoic acid (EPA), is commonly found in marine oils (e.g., fish oils, eggs, krill oil), marine algae and phytoplankton [15]. Quantitatively, DHA is the most abundant PUFA in brain gray matter, providing for the 15–20% of the total fatty acid composition in the frontal areas [16]. DHA is incorporated into cell membrane phospholipids (in exceptionally high levels in the central nervous system) where it covers several important functions and, especially, a structural role influencing the membranes’ fluidity, lateral pressure, bilayer thickness, and surface charge distribution [14]. It can also modify the function of membrane proteins acting as precursors of autocoid signaling molecules (e.g., docosanoids, with anti-inflammatory effects) and as potent activators of a number of gene transcription factors (e.g., peroxisome proliferator activated receptors) [12].

The principal function of ω-3 LC-PUFAs is generally linked to the incorporation of DHA, in exceptionally high levels, in the membranes of the central nervous system. Indeed, DHA is found in significant concentrations in the retinal and synaptic membranes due to its high fluidity [13]. The accretion of DHA in membrane phospholipids cells of different tissues, e.g., erythrocytes (RBC), has been shown to be linked to the diet, mainly fish intake and breastfeeding during infancy, even if another small source of DHA is also produced endogenously by the desaturation and elongation of alpha-linolenic acid (ALA). This biochemical process of conversion is limited by the delta-6 desaturase enzymatic step, which is normally less efficient, and the rate of conversion has been shown to be influenced by the individual asset of the haplotypes, including single-nucleotide polymorphisms (SNPs), related to PUFA metabolism. Indeed, in addition to diet, common polymorphisms in the fatty acid desaturase (FADS) gene cluster seem to have very marked effects on LC-PUFA status [14].

For all these reasons, ω-3 LC-PUFAs are considered to be one of the most important components of cell membranes, including neurons, because they are implicated in the regulation of cross-talk between cells and in the metabolic processes of energy transformation [14]. Additionally, LC-PUFAs are essential components for infant and child neurodevelopment because they are implicated in many neuronal processes (e.g., the regulation of membrane fluidity and gene expression) [14]. LC-PUFAs accumulation in the brain starts during the brain growth spurt in the intrauterine stage and continues up to two years of age [14]. Once high levels of DHA are achieved in the brain, these are maintained during later life, presumably depending on an optimal dietary supply [15].

With respect to the functional effects of LC-PUFA supplementation in infancy, the most accepted developmental effect is an increased rate of visual acuity development [16].

Moreover, the deficiency in or imbalance of ω-3/ω-6 LC-PUFAs has been associated with poorer child neurodevelopment, with a deficit in cognitive domains, for example, language (e.g., verbal fluency) and motor skills (e.g., gross and fine motor abilities) [17,18].

Interestingly, evidence also showed that DHA is required for normal brain development [14]. One possible explanation for the positive correlation between optimal DHA status and cognition may be due to the fact that, during development, one of the most responsive cerebral areas to DHA supplementation is the frontal cortex [19]. The frontal lobes have a key role in executive and higher-order cognitive functions, including sustained attention, planning and problem solving [20], as well as in social, emotional and behavioural development [21]. Therefore, an optimal lipid DHA composition may not only be important during the development and maturation of the brain from pregnancy to childhood/adolescence, but could also contribute to maintaining cognitive efficiency during the entire lifespan and ameliorating the aging of the adult brain [22,23]. Indeed, in the last decade, evidence has accumulated suggesting the neuroprotective properties of ω-3 LC-PUFAs, in particular DHA. Specifically, the primary metabolite responsible for the neuroprotective effect of DHA is the DHA-derivate neuroprotectin D-1 (NPD-1). Under adverse conditions, such as cerebral inflammation, DHA is released from membrane phospholipids to the cytoplasm, and subsequently transformed into NPD-1. This metabolite has the ability to reduce a) the generation of proinflammatory cytokines, b) the formation of β-amyloid peptide, a cytotoxic structure considered to be neurotoxic and an oxidative stress promoter, which disrupts synaptogenesis and induces neuronal apoptosis, and c) the expression of pro-apoptotic genes, which leads to a reduction in neuronal death [24].

Moreover, neuroimaging studies also supported the positive link between ω-3 PUFAs levels and mood functioning. Indeed, a structural Magnetic Resonance Imaging (MRI) study reported that individuals with higher dietary intake levels of ω-3 PUFAs have greater grey matter volume in the anterior cingulate cortex, the right hippocampus, and the right amygdala, which are key areas involved in mood regulation and cognition [25].

Accordingly, recent studies have focused their attention on the potential neuroprotective effects of ω-3 PUFAs in the development of psychiatric illnesses, in particular affective disorders [26]. Although a recent meta-analysis [27] and several trials [28,29,30] reported significant positive effects of ω-3 PUFAs (only EPA or combined EPA+DHA) in ameliorating depressive symptomatology in BD, to date, no RCTs have explored the effect of ω-3 PUFAs on cognitive performance as a primary outcome in BD.

In this context, the present study aims to explore, for the first time to the best of our knowledge, the effects of 12 weeks’ supplementation of DHA on cognitive functions in euthymic BD patients versus healthy controls (HCs). We hypothesized that DHA supplementation would dose-dependently improve cognitive performances in both BD patients and HCs.

## 2. Materials and Methods

### 2.1. Trial Design and Participants

We have conducted an analysis per protocol, single-centre, double-blind randomized controlled trial aiming at evaluating a 12 weeks DHA supplementation versus placebo in 31 euthymic patients with BD type I or type II (13 BD with Omega 3 [mean age 36 ± 12 years; three males and 10 females] and 18 BD with placebo [mean age 50.4 ± 11.3 years; six males and 12 females], all Caucasian) and 15 HCs (seven with Omega 3 [mean age 33.1 ± 12.4 years; four males and three females] and eight with placebo [mean age 39.4 ± 13.9 years; two males and six females], all Caucasian). For this trial, based on the evidence reported by a previous study [31], we were planning to recruit at least 10 participants per group. However, due to the dropout of six HCs before the randomization process, we were not able to achieve this number in the control group. Participants were recruited at the psychiatric research unit of the University Policlinico Hospital of Milan, Italy. The clinical researchers were preliminary trained in the use of the study tools. All patients recruited fulfilled the criteria for a diagnosis of bipolar disorder type I or type II according to the Diagnostic and Statistical Manual of Mental Disorders, 4th edition, text revision (DSM-IV-TR), based on the Structured Clinical Interview for Diagnosis (SCID-I) [32]. The mood state was measured with the Italian version of the Young Mania Rating Scale (YMRS) [33] and the 17-item Hamilton Depression Rating Scale (HDRS-17) [34]. Also, the Global Assessment of Functioning (GAF) [32,35] was obtained from all patients to evaluate symptoms and social functioning. The medications taken by BD patients were monitored every month. All participants were suitable for the trial if a) aged 18-65 years old, b) fulfilled criteria for BD I or II, c) at the time of recruiting were in euthymic phase: YMRS (range 0–2) and HDRS-17 (range 0–7), d) were no more than moderate consumers of EPA and DHA, e) were not pregnant or breast feeding, f) were not severely ill, g) were not known to suffer from medical or other conditions that contraindicated use of the active or placebo supplement (e.g., gastrointestinal conditions, food intolerances, food allergies, special diets).

Exclusion criteria were: (a) history of any axis I psychiatric disorder in first-degree relatives and any current medical problems, (b) patients with co-morbid psychiatric disorders, alcohol or substance abuse within the six months preceding the study, (c) history of traumatic head injury with loss of consciousness (over 15 min), epilepsy or other neurological diseases.

For control subjects, inclusion criteria were: (a) aged 18–65 years old, (b) no DSM-IV axis I disorders, as determined by the SCID-I non-patient version [36], (c) no history of neurologic, or substance-related disorder, (d) not pregnant or breast feeding, (e) not severely ill, (f) not known to suffer from medical or other conditions that contraindicated use of the active or placebo supplement (e.g., gastrointestinal conditions, food intolerances, food allergies, special diets).

Participants were instructed to consume five capsules daily. These five capsules contained a total of either 1250 mg DHA (one pill contains 0,25 mg DHA commercially manufactured from microalgae of the genus Schizochytrium) or 1250 mg corn oil (placebo treatment). Active and placebo capsules were identical in appearance. These capsules were purchased from the Dietetic Metabolic Food Industry, which prepared them specifically for this trial. They were supplied to participants in sealed, child-safe, plastic containers labelled with the participant’s identification group.

Finally, interviewers (Caletti Elisabetta, Prunas Cecilia and Siri Francesca), blinded to each other, administered a cognitive battery and psychiatric scales at baseline (week 0; T0) and after 12 weeks (T2) of supplementation. At each timepoint, participants were required to attend the psychiatric research unit of the University Policlinico Hospital of Milan, Italy. On arrival, participants were first required to provide a blood sample. Following the test batteries, participants were provided with their capsules and instructions on how to take them. Plasma levels of DHA were measured at baseline (T0), after six weeks (T1) and after twelve weeks (T2). However, the delta DHA was calculated based on just the first two timepoints (T0 and T1), since the last timepoint was formed by many missing data. The formula employed for the delta was value at T1– value at T0/value at T0.

Fatty acid levels were expressed as percent of total plasma fatty acids. Participants were free to ask questions throughout the visit and were encouraged to telephone the researchers from home if necessary. Participants were contacted by telephone prior to each visit to confirm their appointment.

All participants gave signed informed consent, after having understood all issues involved in participation in the research. The research was approved by the biomedical Ethics Committee of the University Policlinico Hospital of Milan, Italy. After informed consent was obtained, eligibility was determined and eligible subjects were randomized in a 1:1 fashion to either DHA or placebo (corn oil) in a double blind manner.

### 2.2. Neuropsychological Battery

The cognitive abilities of patients were assessed through a standard neuropsychological battery, the Brief Assessment of Cognition in Affective Disorder (BAC-A) [37,38], a comprehensive test battery developed specifically for patients with mood disorders, which was recently validated in the Italian population [39]. Among cognitive batteries validated in the psychiatric population, BAC-A can be easily administered and scored, and it has been demonstrated to be suitable to estimate global functioning. The BAC-A comprises eight tasks assessing different domains of cognitive function (verbal memory, visuomotor abilities, affective interference, working memory, attention, verbal fluency, problem solving and affective inhibition). Specifically, eight subtests were used, two investigating the emotional domain, which include, (i) Affective Processing Test, which evaluates components of immediate and delayed affective and non-affective memory, and (ii) Emotion Inhibition Test, which measures the ability to suppress an automatic process, like reading, and the irrelevant elaboration of the word’s meaning (affective processing) in a color naming task, and six exploring the cognitive/linguistic domain, including, (iii) List Learning, a measure of verbal learning and memory, iv) Digit Sequencing Task, which evaluates working memory, (v) Token Motor Task, which estimates visuo-motor abilities, (vi) Verbal Fluency, used to evaluate both semantic and phonemic fluency, (vii) Symbol-Coding Task, employed to measure attention and processing speed, (viii) Tower of London, which provides an estimation of problem solving abilities, a subcomponent of executive functions.

### 2.3. Statistical Analysis

Data analyses were performed using R [40]. For exploring the presence of differences between the groups on clinical and sociodemographic variables, we performed a chi–square test (χ^2^), for qualitative variables (i.e., gender), and *t*-tests or F-tests for quantitative variables, using permutation tests to deal with invalid assumptions and the small sample size, corrected by Holm for multiple comparison and by Bonferroni for multiple testing. Permutation tests offer an easy way to compute the sampling distribution for any test statistic, swapping the data labels under the null hypothesis of the exchangeability assumption. Therefore, the permutation tests are one type of non-parametric test that permit to deal with clinical trials with small sample sizes, which can be problematic when trying to achieve the normality assumption.

In order to investigate the differences between the groups in all cognitive tests, we employed multivariate linear model using a hierarchical approach, which can represent the multiple testing problem as a graph structure; the interpretability of the results increases, and we can gain power to discover [41]. To deal with the invalid normality assumption and the small sample size, permutation tests, instead of normal theory tests, for the Multivariate Analyses of Variance (MANOVA) and for the coefficients of the multivariate linear model, were employed.

The variable group considered has four levels: controls who were taking DHA supplementation, patients who were taking DHA supplementation, controls who were taking placebo and patients who were taking placebo. The dependent variables analysed are the delta differences in the test results during time (value at T1—value at T0/value at T0).

First, a MANOVA with all neuropsychological tests variables as dependent variables, as well as group, age, sex and delta of the plasma levels of DHA as covariates, was carried out in order to explore whether the variable “group” was significant. Although we collected the plasma levels of DHA at three timepoints, for the analyses we considered only T0 and T1, since the last timepoint had a large amount of missing data. Then, we divided the neuropsychological tests into emotional and cognitive/linguistic tasks, which have been used as dependent variables in two separate MANOVAs. Since only the MANOVA result regarding the emotional tasks was significant, post-hoc tests were performed to explore the significant main effects of the variable group on only the emotional tasks. Due to the exploratory nature of this study, a formal sample size calculation would have been of little value and therefore it was not performed.

## 3. Results

### 3.1. Socio Demographic and Clinical Variables

Overall, 31 BD patients (13 with Omega 3 and 18 with placebo) and 21 HCs (seven with Omega 3 and eight with placebo) were enrolled. No statistically significant differences were found in any of the socio-demographic variables between patients group vs. HCs, except for age. Specifically, test post-hoc showed that HCs with Omega 3 had lower mean age compared to BD patients with placebo (*t* = -3.2, *p* = 0.05) and BD patients with placebo had higher age compared to BD patients with Omega 3 (*t* = −3.3, *p* = 0.05). Furthermore, significant differences were observed between HCs and BD patients on GAF scores and DHA dosages at baseline. Specifically, we found that BD patients with placebo had lower GAF scores compared to HCs with (*t* = 4.1, *p* = 0.002) or without (*t* = 4.6, *p* = 0.0001) Omega 3. Additionally, BD patients with Omega 3 showed lower GAF scores compared to HCs with placebo (*t* = 3.6, *p* = 0.01). Finally, we observed that HCs with or without Omega 3 had higher levels of DHA at baseline compared to BD with or without Omega 3 (*t* = 3.7, *p* = 0.01). No other significant differences were observed in terms of clinical variables. Please refer to Table 1 for the socio-demographic and clinical details of the whole sample.

### 3.2. Neuropsychological Differences Between the Four Groups

**Figure 1:**
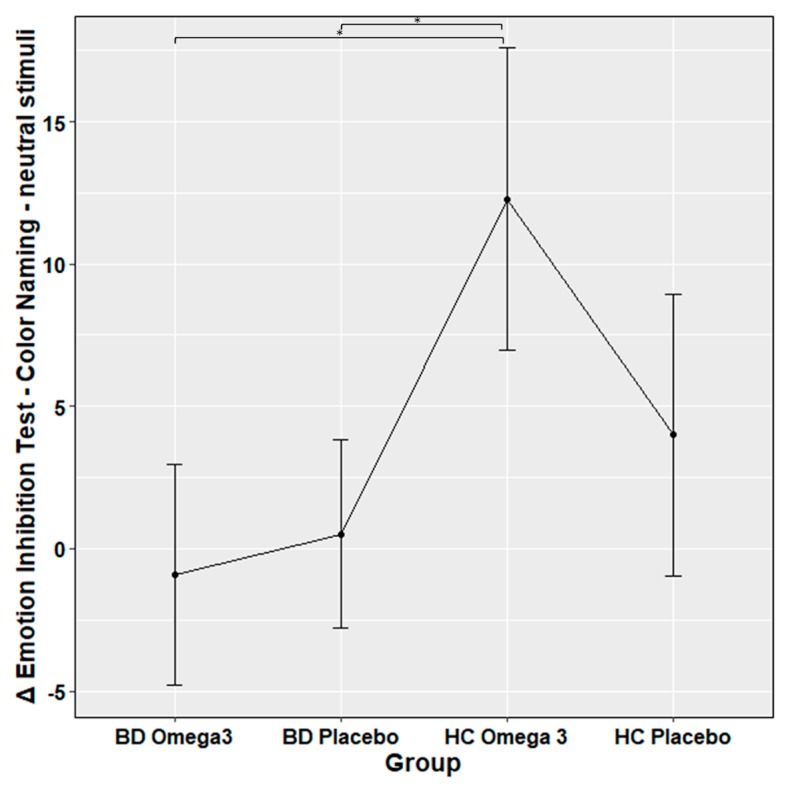
Significant mean differences in Emotion Inhibition Test—Color Naming—neutral stimuli test between bipolar (BD) patients and healthy controls (HCs) with or without Omega 3. The post-hoc tests were calculated on the coefficient of a linear model, corrected for multiple comparisons with Holm method and for multiple testing with Bonferroni correction.

No significant group differences were observed between all the neuropsychological tests between the four groups, except for the emotion inhibition test, where HCs with Omega 3 had higher scores compared to either BD with Omega 3 (*z* = 3.9, *p* = 0.003) or BD with placebo (*t* = 3.7, *p* = 0.005). Table 2 and Figure 1 showed the significant results emerging from the post-hoc analysis.

### 3.3. Correlations Between Tests and Clinical or Sociodemographic Variables in BD Patients

No statistical significant correlations were observed between neuropsychological tests, sociodemographic or clinical variables and DHA supplementation.

## 4. Discussion

Since the role of ω-3 PUFAs has been an object of interest in many psychiatric and neurological disorders, including mood disorders [27], in this study we aimed, for the first time to the best of our knowledge, to evaluate the efficacy of DHA supplementation on cognitive performance in euthymic BD patients and HCs. Interestingly, our results showed that only the group of HCs receiving 12 weeks of DHA supplementation showed an improvement in cognitive performance in the emotion inhibition test from baseline to follow-up, while the group of BD patients did not show any improvement in any tasks of the neuropsychological battery. These results are in agreement with previous literature showing that healthy subjects could have an advantage, linked to the ω-3 PUFAs supplement, especially in cognitive functions like memory, attention and psychomotor speed [42]. More specifically, Fontani et al. (2005) [43] found an improvement in the attention domain, measured by the Go/No-Go and sustained attention tests, in a sample of 49 healthy subjects taking ω-3 PUFAs supplementation. Also, a positive effect on episodic and working memory was found by Stonehouse et al. 2013 [44] in a sample of 228 healthy adult volunteers, mostly female, taking ω-3 PUFAs supplement. In this direction, it seems that our result is also in accordance with a neuroimaging study performed in a sample of HCs, which showed that a higher dietary ω-3 PUFAs intake was related to greater corticolimbic gray matter volume, especially in the subgenual anterior cingulate cortex the right hippocampus and the right amygdala, ultimately suggesting that ω-3 PUFAs might be associated with the integrity of brain structures and especially in the ones involved in cognitive and emotional processing, including memory, mood and affect regulation [45].

Furthermore, from our results it also emerged that, at baseline, our group of HCs had higher DHA serum levels compared to BD patients, further supporting the hypothesis that lower levels of total ω-3 PUFAs, EPA and DHA in patients with affective disorders may play a key role in the pathogenesis and aetiology of affective disorders [46,47]. Indeed, evidence from epidemiological and biochemical studies consistently suggested the presence of a link between BD and the reduced consumption of ω-3 PUFAs [48], ultimately leading to the idea that low intake of Omega 3 might be a risk factor for the development of a mood disorder.

Importantly, several factors can explain why DHA supplementation had ameliorating effects only in the healthy population and not in BD patients. First, the unhealthy lifestyle habits of BD patients. Individuals with BD have poor dietary choices and nutritional habits, since they usually have less than two meals per day and they prefer carbohydrates, sugared beverages, and unhealthy pre-packaged or prepared foods [49]. These behaviors do not support an adequate DHA dietary supply from its principle sources, such as seafood (fish, shellfish, micro- and macroalgae). Moreover, BD patients tend to have a sedentary life and they often smoke and use drugs or alcohol more than patients with any other psychiatric illness [50,51]. All these factors have been found to determine an imbalance between fatty acids metabolism, which may ultimately reduce DHA absorption [52,53,54]. Indeed, it has been reported that the presence of alterations in lipids metabolism in mood disorders lead to changes in cell signaling pathways that could alter neurotransmitter systems [55]. In this regard, a study by McNamara et al. (2010) found that BD patients exhibited selective erythrocyte DHA deficits compared to HCs, which were not only attributed to reductions in desaturase or elongase activity but also to defects in the function of peroxisomes that exert the last step in the biosynthesis of DHA [56]. Similarly, drugs also have a negative impact on the liver and may therefore modify the metabolism of ω-3 PUFAs [57,58]. Therefore, according to this evidence, it might be possible that the lack of results observed in our group of BD patients could be related to the altered ω-3 PUFAs biochemistry caused by unhealthy lifestyle habits, which might have negatively affected our results by masking the effect of DHA supplementation on cognition in BD patients.

Second, the specific clinical characteristics, and especially the presence of multiple hospitalizations and a long duration of untreated illness, in our sample of BD patients might have also had a negative impact on our results. Indeed, it has been reported that the duration of untreated illness might be a negative prognostic factor in many psychiatric conditions, including affective disorders, and may have neurodegenerative effects [59,60]. This hypothesis is further supported by the presence, at baseline, of lower GAF scores in BD patients compared to HCs, despite the presence of comparable educational levels, which also aligned with prior data showing that the number of past episodes is significantly associated with functional outcome in BD patients [61].

Finally, although a 12-week study period for investigating the impact of DHA supplementation has been considered sufficient to observe a clinical change [62], several studies have considered a much longer time period in order to preserve cognitive function during neuroprogression [63,64]. Therefore, it might be plausible that, in order to obtain a significant improvement linked to ω-3 PUFAs treatment, a longer period of exposure may be required.

Importantly, we believe that our results should be considered with caution, since our study suffers from some important limitations. First, the sample size has to be considered a meaningful limitation influencing the statistical power of our analyses. Second, the significant higher age of BD patients with placebo could be, as suggested by previous evidence, a hypothetical reason for a greater cognitive dysfunction in patients with BD compared to HCs [65]. Third, the duration of the trial could represent a potential bias, since this time interval may not be sufficient to achieve significant changes in brain fatty acids composition and, accordingly, to detect influences on cognition in patients with BD. However, the duration of this trial is in line with previous studies on this topic [64,65]. Finally, the missing data of the last measurement of DHA plasma levels could represent a limitation for the complete interpretation of results and statistical analyses.

## 5. Conclusion

In conclusion, this exploratory case-control trial is the first to examine the association between DHA supplementation and cognitive function in euthymic BD patients and HCs. Indeed, although previous studies investigating the effects of ω-3 PUFAs in affective disorders have already been published, their focus was on mood changes or in the investigation of cognition as a secondary outcome [56,57,58]. Overall, our results showed that only the group of HCs receiving 12 weeks of DHA supplementation showed an improvement in cognitive performance in the emotion inhibition test from baseline to follow-up, ultimately suggesting that this non-pharmacological treatment might be effective for ameliorating cognitive performance. However, future studies are needed to (a) optimize ω-3 PUFAs ratios contributing to improve cognitive performances in BD (b) understand the correct time of intervention needed to obtain a significant effect of DHA on cognition, and (c) discover the specific cognitive domains influenced by ω-3 PUFAs supplement.

## Figures and Tables

**Table 1:** Socio-demographic and clinical variables in the four study groups.

	BD-Omega 3 (*n* = 13)	BD-Placebo (*n* = 18)	HCs-Omega 3 (*n* = 7)	HCs Placebo (*n* = 8)	Statistics *	*p*-Value, Bonferroni Corrected	Post-hoc Results (After Correction for Multiple Comparisons with Holm Method)
Age (years), mean ± SD	36 ± 12	50.4 ± 11.3	33.1 ± 12.4	39.4 ± 13.9	F =5.337,	*p* = 0.024	HCs with Omega 3 < BD with placebo BD with placebo > BD with Omega 3
Gender (males/females)	3/10	6/12	4/3	2/6	χ^2^ = 2.67	*p* = 1	
Educational level (years), mean ± SD	14.5 ± 2.93	14.6 ± 3.36	15.6 ± 2.44	14.6 ± 2.26	F = 0.24	*p* = 1	
Race	Caucasian	Caucasian	Caucasian	Caucasian	-	-	-
Age on onset, mean ± SD	26.2 ± 9.68	29.2 ± 11.1	-	-	*t* = 0.75	*p* = 1	
DUI (months), mean ± SD	43.7 ± 59.9	67.9 ± 99.8	-	-	*t* = 0.75	*p* = 1	
No. Hospitalization, mean ± SD	2.38 ± 2.18	1.44 ± 1.69	-	-	*t* = 1.31	*p* = 1	
GAF Total scores, mean ± SD	79.2 ± 9.74	76.7 ± 8.51	91.6 ± 6.48	92.6 ± 4.9	F = 10.78	*p* = 0.008	HCs with Omega 3 = HCs with placebo > BD with Omega 3 = BD with placebo
DHA plasma levels, mean ± SD	2.13 ± 0.803	1.68 ± 0.392	2.59 ± 0.688	1.98 ± 0.039	F = 4.78	*p* = 0.047	HCs with Omega 3 = HCs with placebo > BD with Omega 3 = BD with placebo
Delta DHA plasma levels, mean ± SD	0.578 ± 0.661	0.243 ± 0.371	0.256 ± 0.604	0.270 ± 0.02	F = 1.422	*p* = 1	

BD = Bipolar Disorder; HCs = Healthy Controls; SD = Standard Deviation; DUI = Duration of Untreated Illness; GAF = Global Assessment of Functioning. * The test statistics, i.e., t-tests, ANOVA, chi-squared were calculated under a permutation framework, corrected for multiple comparisons with Holm method and for multiple testing with Bonferroni correction.

**Table 2:** Differences in the emotion inhibition test between bipolar disorder (BD) patients and healthy controls (HCs) with or without Omega 3.

	BD Patients Omega 3 (*n* = 13)	BD Patients Placebo (*n* = 18)	HCs Omega 3 (*n* = 7)	HCs Placebo (*n* = 8)	Statistics*	*p*-Value, Bonferroni Corrected
Emotion Inhibition Test – Color Naming - neutral stimuli, mean accuracy ± SD	0.0008 ± 0.14	0.024 ± 0.13	0.32 ± 0.25	0.121 ± 0.28	HCs-Omega 3 vs. HCs-Placebo; *z* = 2.3 HCs-Omega 3 vs. BD-Omega 3; *z* = 3.9 HCs-Omega 3 vs. BD-Placebo; *z* = 3.7 HCs-Placebo vs. BD-Placebo; *z* = 1.5 HCs-Placebo vs. BD-Omega 3; *z* = 1.5 BD-Omega 3 vs. BD- Placebo; *z* = −0.05	*p* = 0.4 *p* = 0.003 *p* = 0.005 *p* = 1 *p* = 1 *p* = 1
Emotion Inhibition Test – Word Naming - neutral words, mean accuracy ± SD	0.13 ± 0.27	0.86 ± 3.78	0.03 ± 0.23	−0.05 ± 0.096	HCs-Omega 3 vs. HCs-Placebo; *z* = −0.5 HCs-Omega 3 vs. BD-Omega 3; *z* = −0.7 HCs-Omega 3 vs. BD-Placebo; *z* = −1.6 HCs-Placebo vs. BD-Placebo; *z* = −1.2 HCs-Placebo vs. BD-Omega 3; *z* = −0.1 BD-Omega 3 vs. BD-Placebo; *z* = −1.1	*p* = 1 *p* = 1 *p* = 1 *p* = 1 *p* = 1 *p* = 1
Emotion Inhibition Test – Color Naming - neutral words, mean accuracy ± SD	0.013 ± 0.16	−0.026 ± 0.13	0.017 ± 0.09	−0.028 ± 0.07	HCs-Omega 3 vs. HCs-Placebo; *z* = 0.5 HCs-Omega 3 vs. BD-Omega 3; *z* = 0.1 HCs-Omega 3 vs. BD-Placebo; *z* = 0.6 HCs-Placebo vs. BD-Placebo; *z* = 0.05 HCs-Placebo vs. BD-Omega 3; *z* = -0.5 BD-Omega 3vs BD-Placebo; *z* = 0.6	*p* = 1 *p* = 1 *p* = 1 *p* = 1 *p* = 1 *p* = 1
Emotion Inhibition Test – Color Naming - affective words, mean accuracy ± SD	0.0068 ± 0.11	0.018 ± 0.12	0.095 ± 0.27	−0.0013 ± 0.12	HCs-Omega 3 vs. HCs-Placebo; *z* = 1.5 HCs-Omega 3 vs. BD-OMEGA 3; *z* = 1.9 HCs-Omega 3 vs. BD-Placebo; *z* = 1.1 HCs-Placebo vs. BD-Placebo; *z* = −0.6 HCs-Placebo vs. BD-Omega 3; *z* = 0.2 BD-Omega 3 vs. BD-Placebo; *z* = −0.9	*p* = 1 *p* = 1 *p* = 1 *p* = 1 *p* = 1 *p* = 1

BD = Bipolar Disorder; HCs = Healthy Controls; SD = Standard Deviation. * The post-hoc tests were calculated on the coefficient of a linear model, corrected for multiple comparisons with Holm method and for multiple testing with Bonferroni correction.
